# Roles of β-catenin, TCF-4, and survivin in nasopharyngeal carcinoma: correlation with clinicopathological features and prognostic significance

**DOI:** 10.1186/s12935-019-0764-7

**Published:** 2019-02-28

**Authors:** Pei-Ying Jin, Zi-Hui Zheng, Hong-Jie Lu, Jing Yan, Gui-Hong Zheng, Yuan-Lin Zheng, Dong-Mei Wu, Jun Lu

**Affiliations:** 1 Key Laboratory for Biotechnology on Medicinal Plants of Jiangsu Province, School of Life Science, Jiangsu Normal University, No. 101, Shanghai Road, Tongshan District, Xuzhou, 221116 Jiangsu People’s Republic of China; 2 State Key Laboratory Cultivation Base For TCM Quality and Efficacy, School of Medicine and Life Science, Nanjing University of Chinese Medicine, Nanjing, 210023 People’s Republic of China; 3 Emergency Center, The Affiliated Hospital of Xuzhou Medical University, Xuzhou, 221009 People’s Republic of China

**Keywords:** β-Catenin, TCF-4, Survivin, Nasopharyngeal carcinoma, Clinicopathological stages, Survival prognosis

## Abstract

**Background:**

Nasopharyngeal carcinoma (NPC) is a common malignant tumor of the head and neck region with poorly understood progression and prognosis. The present study aims at exploring whether the expression of β-catenin, TCF-4, and survivin affects clinicopathological features and prognostic significance in NPC.

**Methods:**

We enrolled 164 patients with NPC and 70 patients with chronic nasopharyngitis (CNP) in this study. Reverse transcription quantitative polymerase chain reaction (RT-qPCR) and immunohistochemistry (IHC) were conducted to evaluate the expression of β-catenin, TCF-4, and survivin. Spearman’s rank correlation analysis and Pearson correlation analysis were used to measure the correlation of β-catenin, TCF-4, and survivin. Risk factors for prognosis and survival conditions of NPC patients were analyzed by Cox proportional hazards model and Kaplan–Meier curves.

**Results:**

The results obtained revealed that mRNA and protein expression of β-catenin, TCF-4, and survivin was higher in NPC tissues than in CNP tissues. Positive correlations amongst β-catenin, TCF-4, and survivin were identified by Spearman’s rank correlation analysis and Pearson correlation analysis. There was a significant correlation in expression of β-catenin, TCF-4, and survivin with EBV DNA, EBV-VCA-IgA, EBV-EA-IgA, T stage, N stage, and clinicopathological stages. Lower overall survival (OS), distant metastasis-free survival (DMFS), local recurrence-free survival (LRFS), and disease-free survival (DFS) rates were detected in NPC patients with positive expression of β-catenin, TCF-4, and survivin, in contrast to those with negative expression. Cox proportional hazards model demonstrated that β-catenin, TCF-4, and survivin protein positive expression were independent risk factors for OS and DFS of NPC prognosis; there was an evident correlation between clinicopathological stages, TCF-4, and EBV-EA-IgA and OS, DMFS, LRFS, and DFS of NPC.

**Conclusions:**

The aforementioned results indicate that β-catenin, TCF-4, and survivin proteins are highly expressed in NPC, which can be used as factors to predict the malignancy of NPC. In addition, positive expression of β-catenin, TCF-4, and survivin are potential risk factors that lead to an unfavorable prognosis of OS and DFS in NPC patients.

## Background

Nasopharyngeal carcinoma (NPC), frequently occurring in Southern Asia, has been closely linked with tumor angiogenesis [1]. NPC is described as an epithelial malignant disease, with clinical manifestations including nasal bloody discharge (37.9%) and headaches (31.1%), which are the most frequent symptoms in its patients [2, 3]. Epstein–Barr virus (EBV) is detected in all tumor cells except in normal nasopharyngeal epithelium, suggesting the activation of EVB plays an essential role in NPC pathogenesis [4]. NPC is featured by familial, racial and regional aggregations [5]. There’s also higher incidence and mortality associated with NPC in urban areas compared to rural areas [6]. In 2008, NPC was 11th most common malignant tumor in China, with a morbidity of 2.8 in 0.1 million people per year for men, and 1.9 in 0.1 million people per year in women [5]. Radiotherapy is a widely used method for treating NPC, and concurrent chemoradiotherapy (CCRT) can improve outcomes for stage III–IV patients with NPC [7, 8]. However, NPC remains to have poor prognosis due to the nature of NPC tumors, which are prone to late diagnosis, recurrence, and metastasis, leading to increased mortality [9–11]. The 7th version of AJCC Cancer Staging Manual reports that advanced NPC results in an unfavorable 5-year survival rate of 62% and 38% for patients in stage III and stage IV, respectively; therefore, there is an urgent need for effective therapeutic regimens [12].

β-Catenin has been highlighted in literature as an important effector of the wingless/int (Wnt) pathway and crucial mediator of cell proliferation and cell differentiation [13]. Elevated expression of β-catenin along with increased nuclei has been reported to interact with T-cell factor (TCF)-4/lymphoid enhancer factor (LEF) inducing the activation of target genes in order to regulate body tissue, and endoderm [14]. T cell factor (TCF) acts as a special switch capable of suppressing target genes with Groucho repressor, which is directly influenced by Wnt ligands, simultaneously enhancing gene expression once Groucho is converted to β-catenin [15]. In the nucleus, β-catenin binds to TCF-4/LEF-1 to produce a transcription factor complex, which mediates cell proliferation and epithelial–mesenchymal transition (EMT) by influencing genes [16]. Survivin, also known as baculoviral inhibitor of apoptosis repeat-containing 5 (BIRC5), is an important member of the inhibitor of apoptosis (IAP) family and mediator of cell cycles, that has been shown to be up-regulated in multiple cancers, with studies linking it with unfavorable prognostic significance [17, 18]. Previous studies investigating β-catenin/TCF-4 with in relation to various cancers have indicated its potential as a treatment target option for a variety of cancers including NPC [19, 20]. The aim of the current study was to explore the correlations in regard to the expression patterns of β-catenin, TCF-4 and survivin with clinicopathological features as well as their effect on the prognosis of NPC.

## Materials and methods

### Ethics statement

This study was approved by the Ethics Committee of the Affiliated Hospital of Xuzhou Medical University, and all study subjects provided written informed consents prior to the study.

### Study subjects

A total of 164 NPC patients (117 males and 47 females) within the ages of 24–70 years, with a mean age of (45.3 ± 9.2) years and a median age of 45.0 years, that received treatment at the Oncology Department of the Affiliated Hospital of Xuzhou Medical University between January 2009 and December 2010 were selected for the present study. The inclusion criteria included patients whose NPC diagnosis had been pathologically approved, patients who haven’t received any treatment in the form of chemotherapy, biotherapy, or radiotherapy, and patients who were proven to have no distant metastasis. The normal control group was comprised of paraffin-embedded chronic nasopharyngitis (CNP) tissue specimens that were obtained from the biopsies of 70 patients (58 males and 12 females, with a mean age of (43.9 ± 8.6) years, and median age of 43.5 years) with CNP during the same period. The above paraffin-embedded pathology specimens were well preserved. There was no significant difference regarding age and gender among the NPC patients and patients with CNP (all *P *> 0.05).

### Reverse transcription quantitative polymerase chain reaction (RT-qPCR)

The total RNA of tissue specimens was extracted in accordance with the instructions of Trizol Reagent kit (Solarbio, Shanghai, China). RNA concentration was calculated using the ratio of OD260/280, which was detected by ultraviolet spectrophotometer. The extracted RNA was stored at − 80 °C for future use. Primers of β-catenin, TCF-4, and survivin were designed using Premier 5.0 software (Premier Biosoft International, Palo Alto, CA, USA) in compliance with gene sequences published by GenBank database (Table 1), synthesized by Sangon Biotech Co., Ltd. (Shanghai, China). Reverse transcription PCR of total RNA was performed in accordance with instructions provided by Reverse Transcription System A3500 (Promega Corporation, Madison, WI, USA). A two-step PCR was conducted as follows: 40 cycles from initial denaturation for 3 min at 95 °C, denaturation for 30 min at 95 °C, then 60 s at 60 °C, 1 min at 72 °C, followed by 5 min of denaturation at 72 °C. The reaction system included the following: 12.5 μl of Premix Ex Taq or SYBR Green Mix, 1 μl of Forward Primer, 1 μl of Reverse Primer, 4 μl of DNA template, and 6.5 μl of ddH2O. Glyceraldehyde-3-phosphate dehydrogenase (GAPDH) was regarded as the internal control. A melting curve was employed to improve the reliability of the PCR results, and cycle threshold (Ct) values (inflection point of amplification curve) were used for the calculation of the relative expression of target genes using the 2^−∆∆Ct^ method [21].

**Table 1:** Primer sequences for reverse transcription quantitative polymerase chain reaction

Genes	Primer sequences
β-catenin	Forward: 5′-GCTGATTTGATGGAGTTGGA-3′
	Reverse: 5′-TCA GCT ACTTGTTCTTGAGTGAA-3′
TCF-4	Forward: 5′-CGAGGGTGATGAGAACCTGC-3′
	Reverse: 5′-CCCATGTGATTCGATGCGT-3′
Survivin	Forward: 5′-ATGGGTGCCCCGACGTTGC-3′
	Reverse: 5′-TCAATCCATGGCAGCCAGCTG-3′
GAPDH	Forward: 5′- ACCCAGAAGACTGTGGATGG-3′
	Reverse: 5′-GGAGACAACCTGGTCCTCAG-3′

*TCF-4* T-cell factor-4, *GAPDH* glyceraldehyde-3-phosphate dehydrogenase

### Immunohistochemistry (IHC)

Formalin-fixed paraffin-embedded tissue sections were sliced with the thickness of 3 μm. Then a 10-min incubation was followed by routine deparaffinization and an addition of 3% H_2_O_2_ (Solarbio, Shanghai, China). Next, the sections were boiled in citric acid buffer for 10 min, and then blocked in serum for another 10 min for the removal of the supernatant. Primary antibodies of rabbit anti-human TCF-4 (dilution of 1: 500), mouse anti-human survivin (dilution of 1: 100) and rabbit anti-human β-catenin (dilution of 1: 500) (Santa Cruz Biotechnology, Santa Cruz, CA, USA) were added and incubation was carried out overnight at 4 °C. Afterwards, Phosphate-buffered saline (PBS) was added as the negative control (NC), replacing the primary antibody. Biotinylated secondary antibodies (Solarbio, Shanghai, China) were added into sections successively followed by incubation for 1 h at room temperature. The sections were washed three times (each time for 5 min) using PBS before chromogen was carried out with chromogenic reagents. Subsequently, counterstaining was conducted with hematoxylin (Solarbio, Shanghai, China) prior to dehydration, permeability, and mounting. Afterwards, sections were observed under a fluorescence microscope.

Pale brown or red particles observed by immunohistochemical analysis in β-catenin protein cytoplasm or nuclei were regarded as positive cells; and brown yellow or yellow particles observed in TCF-4 and survivin nuclei were defined as positive cells. The criterion of cell positive expression was the percentage of the positive cell count in the total tumor cell count. Staining intensity criteria were as follows: 0 presents as negative, 1 presents as weak positive, 2 presents as positive, and 3 presents as strongly positive. For the number of positive cells: 0 presents as 0–10%, 1 presents as 11–25%, 2 presents as 26–50%, and 3 presents as over 50%. The final score was obtained from the sum of staining intensity and the number of positive cells. A score of 0–2 was considered negative, and 3–6 was considered positive for IHC staining. In terms of the positively expressed sections, 5 different fields of high magnification were selected for observation under optical microscopy (with the same magnification), and the gray value of immune products was determined by HPIAS-1000. A lower level of gray value indicated stronger staining intensity, and a higher level displayed a weaker staining intensity.

### Postoperative follow-up and survival analysis

Follow-ups were performed through clinic cases, telephone communication, rehospitalization, and visits. The follow-up was conducted for 3-month beginning from the date of radiotherapy until standard discharge was achieved with a last visit date of October 30, 2015. The overall survival (OS), local recurrence-free survival (LRFS), distant metastasis-free survival (DMFS), and disease-free survival (DFS) conditions were major concerns in the follow-up. The OS was the duration from the date of each patient’s random assignment to the date of death from any cause, or the censoring of the patient at the date of the last follow-up; LRFS was the first local recurrence time after radiotherapy; DMFS time was measured from the first distant metastasis time after radiotherapy; DFS time was measured from the time of tumor recurrence, metastasis, and death after radiotherapy. Kaplan–Meier curves were plotted to evaluate survival analysis. The prognosis and risk factors in NPC patients were analyzed though multivariate analysis on Cox proportional hazards.

### Statistical analysis

SPSS 21.0 software SPSS (Version X; IBM, Armonk, NY, USA) was employed for data analysis. Measurement data were expressed by mean value ± standard deviation. Comparisons among gene mRNA expression were conducted with the use of the *t*-test. Enumeration data were represented by number or rate. Comparisons between groups were analyzed by Chi square test. Correlations among β-catenin, TCF-4 and survivin were observed using Pearson correlation analysis. Correlations among β-catenin, TCF-4 and survivin expression were measured using Spearman’s rank correlation analysis. The univariate analysis on prognosis of NPC was determined by Kaplan–Meier curves and the multivariate analysis on prognosis of NPC was determined by Cox regression method. A *P*-value less than 0.05 suggested a statistically significant value.

## Results

### Expression of β-catenin, TCF-4 and survivin mRNA in NPC tissues is higher than those in CNP tissues

**Fig. 1:**
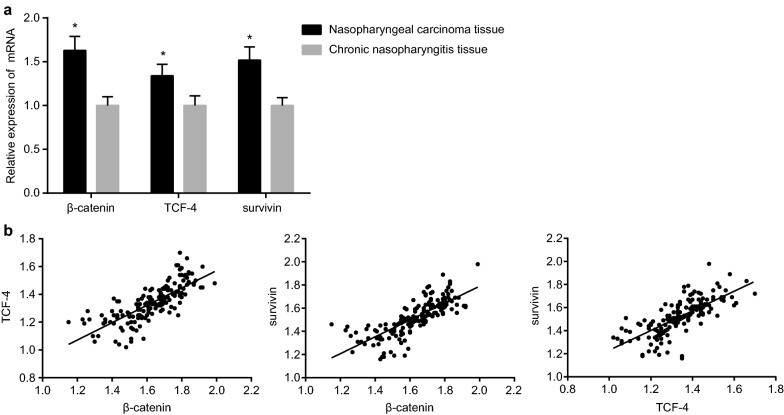
Comparisons (**a**) and correlations (**b**) on expression of β-catenin, TCF-4 and survivin mRNA in NPC tissues and CNP tissues. *P < 0.05 compared with CNP tissues; *TCF-4* T-cell factor-4, *NPC* nasopharyngeal carcinoma, *CNP* chronic nasopharyngitis

The results from RT-qPCR revealed that expression of β-catenin, CF-4 and survivin mRNA in NPC tissues was significantly higher than those in CNP tissues (all *P *< 0.05) (Fig. 1a). Pearson correlation analysis showed that there existed a positive correlation between β-catenin and TCF-4 (r = 0.767, *P* < 0.01), β-catenin and survivin (r = 0.775, *P *< 0.01), and TCF-4 with survivin (r = 0.739, *P* < 0.01) (Fig. 1b). In summary, β-catenin is positively correlated with TCF-4 and survivin, and TCF-4 is positively correlated with survivin in NPC tissues.

### β-Catenin, TCF-4, and survivin proteins are expressed at a higher level than in NPC tissues

**Fig. 2:**
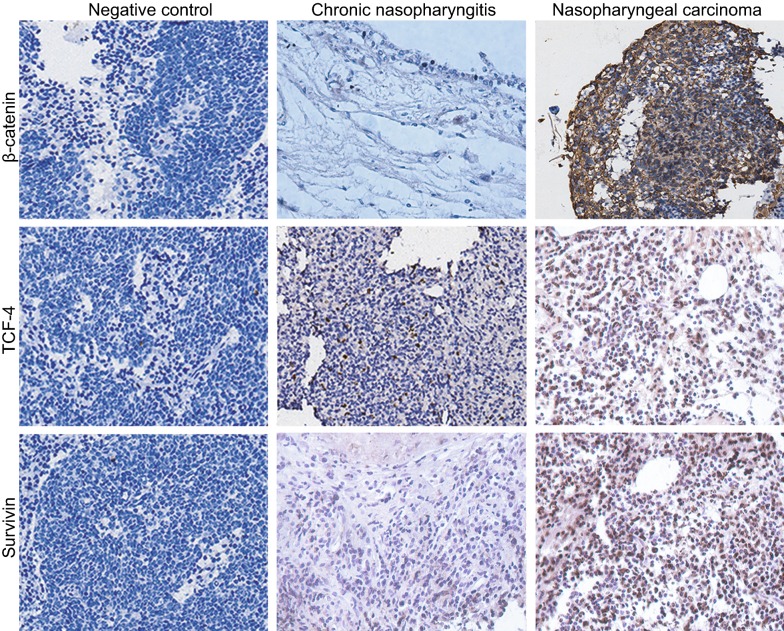
Immunohistochemical analysis on expression of β-catenin, TCF-4 and survivin proteins in the NPC tissues and CNP tissues (SP, ×200). *TCF-4* T-cell factor-4, *NPC* nasopharyngeal carcinoma, *SP* streptavidin-perosidase, *CNP* chronic nasopharyngitis

IHC results found that β-catenin protein is mainly located in nuclei and cell membranes, which was identified by the presence of pale brown or red particles in cytoplasm which indicated positive reaction. The protein’s positive expression rates in NPC tissues and CNP tissues were 0.73 and 0.14 respectively, with statistically significant differences (*P *< 0.05). TCF-4 protein is mainly located in the nuclei, with brown or yellow particles indicating as a positive reaction. The positive expression rates of TCF-4 protein in NPC tissues and CNP tissues were 0.71 and 0.13, respectively (*P *< 0.05). Survivin protein is also mainly located in the nuclei, with brown or yellow particles as a positive reaction. Survivin protein’s positive expression rates in NPC tissues and CNP tissues were 0.70 and 0.11, respectively (*P *< 0.05) (Table 2, Fig. 2). The above results highly indicated that protein expression rates of β-catenin, TCF-4 and survivin were higher in NPC tissues in comparison to that of the CNP tissues.

**Table 2:** Expressions of β-catenin, TCF-4 and survivin protein in NPC tissues and CNP tissues

Group	β-catenin		*P*	TCF-4		*P*	Survivin		*P*
	Positive	Rate		Positive	Rate		Positive	Rate	
NPC tissues (n = 164)	120	0.73	< 0.001	117	0.71	< 0.001	114	0.70	< 0.001
CNP tissues (n = 70)	10	0.14		9	0.13		8	0.11	

*TCF-4* T-cell factor-4, *NPC* nasopharyngeal carcinoma, *CNP* chronic nasopharyngitis

### β-Catenin is positively correlated with TCF-4 and survivin, and TCF-4 is positively correlated with survivin in NPC tissues

According to Spearman’s rank correlation analysis, 99 cases of positive expression and 26 cases of negative expression of β-catenin and TCF-4 were obtained in a total of 164 cases of NPC tissues, which was suggestive of a positive correlation between β-catenin and TCF-4 (r = 0.408, *P *< 0.01). In addition, a positive correlation of β-catenin and survivin (r = 0.376, *P *< 0.01) was determined when 96 cases of positive expression and 26 cases of negative expression were observed. Furthermore, 106 cases of positive expression and 39 cases of negative expression of TCF-4 and survivin were found in 164 cases of NPC tissues, indicating a positive correlation between TCF-4 and survivin (r = 0.723, *P *< 0.01) (Table 3). These findings suggest that there exists a positive correlation between β-catenin and TCF-4 and survivin, and that TCF-4 is also positively correlated with survivin in NPC tissues.

**Table 3:** Correlation of β-catenin, TCF-4 and survivin expressed in NPC tissues

	TCF-4		Survivin	
	−	+	−	+
β-catenin				
−	26	18	26	18
+	21	99	24	96
Survivin				
−	39	8	–	–
+	8	106	–	–

*TCF-4* T-cell factor-4, *NPC* nasopharyngeal carcinoma, *+* positive expression, *−* negative expression

### Protein expression of β-catenin, TCF-4 and survivin is associated with EBV DNA, EBV-VCA-IgA, EBV-EA-IgA, T stage, N stage and clinicopathological stages in NPC

In all NPC evaluated tissue specimens, there was a significant correlation between the expression of β-catenin and EBV DNA, EBV-VCA-IgA, EBV-EA-IgA, T stage, N stage, and clinicopathological stages of NPC (all *P *< 0.05), with no significant correlations in gender, age, smoking history, family history, and histological classification (all *P *> 0.05). Expression of TCF-4 protein significantly correlated with EBV DNA, EBV-VCA-IgA, EBV-EA-IgA, T stage, N stage, and clinicopathological stages (all *P *< 0.05), however, there were no significant correlations between NPC patients’ gender, age, smoking history, family history, and histological classification (all *P *> 0.05). Moreover, expression of survivin protein share the same correlation with clinicopathological features of NPC (Table 4). These findings suggest that expression of β-catenin, TCF-4, and survivin protein is closely correlated with EBV DNA, EBV-VCA-IgA, EBV-EA-IgA, T stage, N stage, and clinicopathological stages.

**Table 4:** Correlations of expressions of β-catenin, TCF-4 and survivin with clinicopathological features of NPC

Clinicopathological features	n	β-catenin				TCF-4				Survivin			
		−	+	Positive rate	*P*	−	+	Positive rate	*P*	−	+	Positive rate	*P*
Age													
≤ 45	86	27	59	0.69	0.166	26	60	0.7	0.64	29	57	0.66	0.345
> 45	78	17	61	0.78		21	57	0.73		21	57	0.73	
Gender													
Male	117	28	89	0.76	0.186	32	85	0.73	0.559	32	85	0.73	0.169
Female	47	16	31	0.66		15	32	0.68		18	29	0.62	
Smoking history													
Yes	107	24	83	0.78	0.082	32	75	0.7	0.628	32	75	0.7	0.825
No	57	20	37	0.65		15	42	0.74		18	39	0.68	
Family history													
Yes	95	23	83	0.87	0.193	27	68	0.72	0.937	26	69	0.73	0.309
No	69	21	48	0.7		20	49	0.71		24	45	0.65	
EBV DNA													
Positive	116	15	101	0.87	<0.001	24	92	0.79	0.001	27	89	0.77	0.002
Negative	48	29	19	0.40		23	25	0.52		23	25	0.52	
EBV-VCA-IgA													
Positive	111	21	90	0.81	0.001	22	89	0.80	<0.001	28	83	0.75	0.034
Negative	53	23	30	0.57		25	28	0.53		22	31	0.58	
EBV-EA-IgA													
Positive	64	4	60	0.94	<0.001	5	59	0.92	<0.001	7	57	0.89	<0.001
Negative	100	40	60	0.60		42	58	0.58		43	57	0.57	
T stage													
T1	28		4	0.14	<0.001	18	10	0.36	<0.001	16	12	0.43	0.004
T2	52	16	36	0.69		12	40	0.77		19	33	0.63	
T3	48	2	46	0.96		15	33	0.69		13	35	0.73	
T4	36	2	34	0.94		2	34	0.94		2	34	0.94	
N stage													
N0	27	25	2	0.07	<0.001	20	7	0.26	<0.001	16	11	0.41	0.006
N1	41	17	24	0.59		11	30	0.73		18	23	0.56	
N2	47	1	46	0.98		9	38	0.81		8	39	0.83	
N3	49	24	48	0.98		7	42	0.86		8	41	0.84	
Clinicopathological stages													
I	19	16	3	0.16	<0.001	13	6	0.32	0.002	12	7	0.37	0.021
II	22	10	12	0.55		13	9	0.41		11	11	0.5	
III	35	10	25	0.71		8	27	0.77		10	25	0.71	
IVa	49	6	43	0.88		8	41	0.84		12	37	0.76	
IVb	39	2	37	0.95		5	34	0.87		5	34	0.87	
Histological classification													
Differentiated non-keratinized	113	27	86	0.76	0.207	31	82	0.73	0.606	32	81	0.72	0.370
Undifferentiated non-keratinized	51	17	34	0.67		16	35	0.69		18	33	0.65	

The standard of family history of nasopharyngeal carcinoma was at least two or more NPC patients in the two generation, and is the first-degree relatives. The standard of smoking history was to smoke one cigarette every day for 6 months or more

*TCF-4* T-cell factor-4, *NPC* nasopharyngeal carcinoma, *T* tumor, *N* node, *+* positive expression, *−* negative expression

### OS, DMFS, LRFS, and DFS rates of β-catenin, TCF-4 and survivin positive expression groups present lower levels when compared with β-catenin negative expression groups

**Fig. 3:**
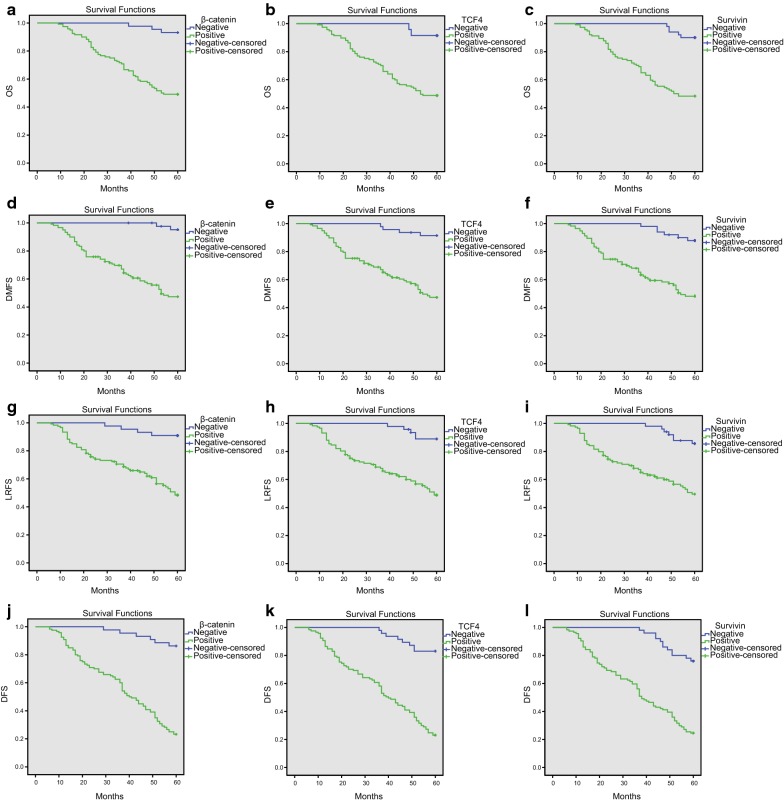
Kaplan-Meier survival curves of β-catenin, TCF-4 and survivin for OS, DMFS, LRFS and DFS conditions of NPC patients. **a** OS of NPC patients with β-catenin expression; **b** OS of NPC patients with TCF-4 expression; **c** OS of NPC patients with survivin expression; **d** DMFS rates of NPC patients with β-catenin expression; **e** DMFS rates of NPC patients with TCF-4 expression; **f** DMFS rates of NPC patients with survivin expression; **g** LRFS rates of NPC patients with β-catenin expression; **h** LRFS rates of NPC patients with TCF-4 expression; **i** LRFS rates of NPC patients with survivin expression; **j** DFS rates of NPC patients with β-catenin expression; **k** DFS rates of NPC patients with TCF-4 expression; **l** DFS rates of NPC patients with survivin expression; *TCF-4* T-cell factor-4, *NPC* nasopharyngeal carcinoma, *OS* overall survival, *DMFS* distant metastasis-free survival, *LRFS* local recurrence-free survival, *DFS* disease-free survival

A Kaplan–Meier survival analysis was performed to identify correlations of expression between β-catenin, TCF-4, and survivin with NPC patient prognosis. All NPC patient follow-ups were performed for 9–60 months with a mean time of 49.2 months. The survival rate was calculated beginning from the end date of radiotherapy. Based on the results, a total of 64 out of 164 patients died (39% mortality); 28 died from local recurrence (accounting for 43.8% of the total deaths), 30 from distant metastasis (accounting for 46.9% of the total deaths), and 6 from other causes (accounting for 9.4% of the total deaths). Kaplan–Meier survival curves showed that the OS, DMFS, LRFS, and DFS rates of the β-catenin positive expression group were 49.2%, 64.2%, 62.5%, and 37.5%, respectively; a lower level compared to the β-catenin negative expression group (*P* < 0.05). OS, DMFS, LRFS, and DFS rates in the TCF-4 positive expression group were 48.7%, 65.0%, 62.4%, and 39.3%, respectively; a lower level compared to the TCF-4 negative expression group (*P* < 0.05). OS, DMFS, LRFS, and DFS rates in the survivin positive expression group were 48.2%, 64.0%, 62.3%, and 39.5%, respectively; a lower level compared to the survivin negative expression group (*P* < 0.05) (Fig. 3). In summary, the aforementioned findings found that there were lower levels in OS, DMFS, LRFS, and DFS rates in β-catenin, TCF-4, and survivin positive expression groups compared to the β-catenin, TCF-4 and survivin negative expression groups in NPC patients.

### β-Catenin, TCF-4, survivin, clinicopathological stages, TCF-4, and EBV-EA-IgA are risk factors in NPC patient prognosis

Multivariate analysis was conducted in order to evaluate risk factors in NPC patient prognosis. Age, gender, smoking history, family history, histological classification, EBV DNA, EBV-VCA-IgA, EBV-EA-IgA, T stage, N stage, clinicopathological stages, and the protein expression of β-catenin, TCF-4 and survivin was analyzed by Cox proportional hazards model. The results were as follows: EBV-VCA-IgA, EBV-EA-IgA, clinicopathological stages, β-catenin, TCF-4, and survivin protein expression was independent risk factors for OS of NPC; EBV-EA-IgA, clinicopathological stages, β-catenin, TCF-4, and survivin were independent risk factors for DMFS of NPC; Family history, N stage, TNM stage, TCF-4, EBV DNA, and EBV-EA-IgA as independent risk factors for LRFS of NPC; EBV DNA, EBV-VCA-IgA, EBV-EA-IgA, clinicopathological stages, β-catenin, TCF-4, and survivin were independent prognostic risk factors for DFS of NPC (all *P* < 0.05) (Table 5). In summary, these findings suggest that positive expression of β-catenin, TCF-4, and survivin proteins is independent risk factors for OS and DFS of NPC prognosis, while clinicopathological stages and TCF-4 and EBV-EA-IgA are significantly related to OS, DMFS, LRFS, and DFS of NPC.

**Table 5:** Cox proportional hazards model for regression analysis on risk factors for prognosis of NPC patients

Features	B	SE	Wald	Sig.	Exp (B)	95.0% CI for Exp (B)	
OS							
Clinicopathological stages	1.508	0.281	28.846	< 0.001	4.517	2.605	7.832
β-catenin	1.738	0.667	6.787	0.009	5.687	1.538	21.030
TCF4	1.515	0.619	5.993	0.014	4.548	1.353	15.293
Survivin	1.499	0.555	7.289	0.007	4.479	1.508	13.300
EBV-VCA-IgA	2.607	0.779	11.190	0.001	13.562	2.944	62.482
EBV-EA-IgA	2.476	0.486	25.986	< 0.001	11.890	4.590	30.803
DMFS							
Clinicopathological stages	1.873	0.283	43.663	< 0.001	6.508	3.734	11.343
β-catenin	2.334	0.753	9.610	0.002	10.321	2.359	45.146
TCF4	2.145	0.562	14.544	< 0.001	8.538	2.836	25.707
EBV-EA-IgA	0.766	0.349	4.806	0.028	2.151	1.085	4.268
LRFS							
Family history	1.181	0.282	17.512	< 0.001	3.257	1.873	5.663
N stage	0.736	0.195	14.232	< 0.001	2.087	1.424	3.058
Clinicopathological stages	1.230	0.219	31.463	< 0.001	3.421	2.226	5.257
TCF4	1.754	0.521	11.323	0.001	5.776	2.080	16.041
EBV DNA	1.393	0.464	9.008	0.003	4.028	1.622	10.004
EBV-EA-IgA	1.162	0.343	11.497	0.001	3.198	1.633	6.261
DFS							
Clinicopathological stages	1.639	0.203	65.080	< 0.001	5.150	3.458	7.669
β-catenin	1.232	0.484	6.470	0.011	3.429	1.327	8.863
TCF4	1.635	0.540	9.163	0.002	5.131	1.780	14.793
Survivin	1.039	0.464	5.011	0.025	2.826	1.138	7.016
EBV DNA	1.396	0.351	15.780	< 0.001	4.039	2.028	8.042
EBV-VCA-IgA	1.467	0.335	19.171	< 0.001	4.336	2.249	8.362
EBV-EA-IgA	1.239	0.275	20.293	< 0.001	3.452	2.014	5.919

*TCF-4* T-cell factor-4, *NPC* nasopharyngeal carcinoma, *T* tumor, *N* node, *SE* standard error, *Sig.* significance, *95% CI* 95% confidence interval, *OS*, overall survival; DMFS, distant metastasis-free survival; LRFS, local recurrence-free survival; DFS, disease-free survival

## Discussion

Nasopharyngeal carcinoma, has been recognized as the most common form of head and neck carcinoma (HNC) and has high prevalence in Southeastern Asia. Although NPC is known to have an aggressive progression and unfavorable prognosis, the underlying factors that contribute to this property of the disease remain unclear [22, 23]. Numerous studies have established the pivotal roles of β-catenin and TCF-4 as essential oncogenes of tumorigenesis, differentiation, and proliferation of tumor cells in the Wnt signaling pathway [19, 24]. Based on the data collected and evaluated in this current study, there was a high expression of β-catenin, TCF-4, and survivin in NPC tissues, which was then linked to T stage, N stage, and clinicopathological stages of NPC tissues. In addition, β-catenin, TCF-4, and survivin, were positively correlated with each other, and along with clinicopathological stages, are risk factors affecting the prognosis of NPC patient.

Based on the results obtained from RT-qPCR and immunohistochemical analysis, there was a significant increase in expression of β-catenin, TCF-4, and survivin mRNA and protein are in NPC tissues in comparison to that of the CNP tissues. Moreover, there was a positive correlation between β-catenin, TCF-4, and survivin. An interesting finding identified the hyper-activation or aberrant activation of β-catenin/TCF-4 as one of the most common abnormalities of signaling in multiple kinds of carcinomas [25, 26]. A previous study also found that β-catenin exhibited higher expression in NPC tissues than in CNP tissues, while the knockdown of β-catenin resulted in the suppression of NPC cell growth, migration and invasion, while inducing cell apoptosis [27]. As an essential member of the Wnt signaling pathway, TCF-4 is known for its involvement in the activation of the Wnt target genes through its interaction with β-catenin. Furthermore, since the Wnt signaling pathway plays a critical role in progression of tumor cells, TCF-4 expression could influence cancer cells in their cellular activities and development of lung cancer [28]. β-catenin only interacts with the TCF family in regards to transcription factors, but can also trans-activate the downstream target genes including cyclin D1 and c-Myc, consequently mediating cell functions including survival, proliferation, migration, and differentiation [29, 30]. Survivin is a major contributor in modulating mitotic activity and apoptosis, and multiple studies have demonstrated its higher expression in malignancies and almost undetectable expression in normal tissues [31, 32]. NPC cells and tissues presented with elevated expression of survivin when compared to normal nasopharyngeal epithelium, and its overexpression was highly correlated with clinical stage III/IV, T stage T3/T4, lymph node metastasis (N1–3), and low 5-year OS rate [18].

Clinicopathological and prognostic significances of β-catenin, TCF-4, and survivin in NPC patients were analyzed using Chi square test, Kaplan–Meier curves, and Cox proportional hazards model. Based on the findings, EBV DNA, EBV-VCA-IgA, EBV-EA-IgA, T stage, N stage, and clinicopathological stages in NPC tissues were correlated with expression of β-catenin, TCF-4 and survivin. The two kinds of EBV-related antibodies, EA-IgA and IVCA-IgA, have been widely applied in the diagnosis and in the prediction of the prognosis of NPC [33]. It has previously been reported that the activation of the β-catenin by EBV may contribute to the lymphoproliferation characteristic of EBV-infected B-cells [34]. Moreover, TCF-4 is a member of the TCF/LEF family of transcription factors, which interacts with β-catenin to mediate Wnt signaling in vertebrates [35]. Furthermore, there was an evident association between the expression of survivin and the histological grade, clinical stage, clinical outcome, as well as survival rate, which could be used as a defining diagnostic marker for cancers [36]. A previous study also suggested that overexpression of survivin is always present in early-stage lung cancer, indicating that survivin may play a role in lung tumorigenesis [37]. The aforementioned findings were all indicatives of the differences that exist among the EBV-DNA load in different stages of NPC, and that the load of advanced NPC was significantly higher than that of early NPC, suggesting that there was a correlation between the load and tumor stage.

In addition, lower OS, DMFS, LRFS and DFS rates were found in positive expression of β-catenin, TCF-4, and survivin. Multivariate analysis identified clinicopathological stages, β-catenin, TCF-4, and survivin as risk factors affecting the prognosis of NPC. A study conducted by Takahashi et al. suggested that there were high levels of β-catenin expression observed in colon cancer, particularly in invasion fronts, and that the accumulation of β-catenin in nuclei correlated with the development of adenoma to invasive carcinoma [38]. The in vitro and in vivo study conducted by Lee et al. suggest that there was an overexpression of cytoplasmic/nuclear β-catenin in tumor cells extracted from primary HNC tissues, and the overexpression enhanced proliferation of HNC cells [39]. There have been a number of studies suggesting that elevated expression of β-catenin predisposes HNC patients to unfavorable prognosis and low survival rates [40]. Previous studies observe that TCF-4 could influence the progression of multiple cancers including lung cancer, hepatocellular cancer, and breast cancer [25, 41, 42]. Survivin is a notable regulator in cell cycle distribution and cellular apoptosis, as well as a risk factor for undesirable prognosis [43]. In addition, survivin, which has been found to be overexpressed in 80% of HNC and oral cancers, is commonly elevated in multiple kinds of carcinomas including NPC, with poor differentiation and metastasis, and is associated with low OS rates [44–46]. These findings are consistent with the multivariate analysis results of this study. Alajez et al. suggest that overexpression of survivin is linked to an unfavorable treatment outcome of NPC patients, and the inhibition of survivin could promote apoptosis and delay tumor aggression in NPC tissues [47]. Some reports indicate that the EBV DNA determination in serum or plasma shows high specificity and sensitivity in NPC diagnosis [48]. Interestingly, a previous study revealed that there is a correlation between patients with NPC serum expression levels of EA-IgA and VCA-IgA and TNM stage [49]. Zhang et al. observed that tumors positive for NF-κB are related to an increased relapse potential, decreased OS, and poor DFS and OS in NPC [50].

## Conclusions

In conclusion, the results obtained from the present study indicated that overexpression of β-catenin, TCF-4, and survivin could potentially play a role as predictors of progression of NPC, and their positive expression may be risk factors for unfavorable prognosis of patients with NPC. However, due to insufficient sample size, there was a high percentage of patients with family history of NPC that were included in our study. Therefore, further large-scale investigations with multiple centers are required to better illustrate and understand the clinical potentials of the expression of β-catenin, TCF-4 and survivin in NPC.

## Abbreviations

NPC: nasopharyngeal carcinoma
RT-qPCR: reverse transcription quantitative polymerase chain reaction
IHC: immunohistochemistry
OS: overall survival
DMFS: distant metastasis-free survival
LRFS: local recurrence-free survival
DFS: disease-free survival
HNC: head and neck carcinoma
EBV: Epstein–Barr virus
CCRT: concurrent chemoradiotherapy
TCF: T cell factor
LEF: lymphoid enhancer factor
EMT: epithelial–mesenchymal transition
BIRC: baculoviral inhibitor of apoptosis repeat-containing
IAP: inhibitor of apoptosis
PBS: phosphate-buffered saline
NC: negative control
